# An investigation of health insurance policy and behavior in a virtual environment

**DOI:** 10.1371/journal.pone.0248784

**Published:** 2021-04-06

**Authors:** J. Dustin Tracy, Kevin A. James, Hillard Kaplan, Stephen Rassenti

**Affiliations:** Economic Science Institute, Chapman University, Orange, CA, United States of America; Brigham Young University, UNITED STATES

## Abstract

We introduce a new experimental approach to measuring the effects of health insurance policy alternatives on behavior and health outcomes over the life course. In a virtual environment with multi-period lives, subjects earn virtual income and allocate spending, to maximize utility, which is converted into cash payment. We compare behavior across age, income and insurance plans—one priced according to an individual’s expected cost and the other uniformly priced through employer-implemented cost sharing. We find that 1) subjects in the employer-implemented plan purchased insurance at higher rates; 2) the employer-based plan reduced differences due to income and age; 3) subjects in the actuarial plan engaged in more health-promoting behaviors, but still below optimal levels, and did save at the level required, so did realize the full benefits of the plan. Subjects had more difficulty optimizing choices in the Actuarial treatment, because it required more long term planning and evaluating benefits that compounded over time. Contrary, to model predictions, the actuarial priced insurance plan did not increase utility relative to the employer-based plan.

## Introduction

US health care costs consume 17.5% of GDP [1], or nearly 10,000 per person, close to twice that of other developed nations [2, 3]. At the household level, these costs can result in bankruptcies and other financial hardships [4]. Yet, even with this vast spending, there are still unmet medical needs. 64% of Americans say that have avoided or delayed medical care in the last year due to expected costs [5]. This combination of high costs and unmet need have led to on-going debate about healthcare and insurance reform.

Much of the debate regarding health insurance policy concerns individual freedom and responsibility versus universal coverage and reducing health disparities [6]. Proponents of universal coverage argue that it improves the average health of the population, decreases disparities in health outcomes within the population, and generates efficiencies by eliminating the transactions costs associated with private insurance billing systems. Proponents of systems emphasizing individual responsibility argue two points: first, that such plans allow people to exert their own priorities in health decisions regarding how much and what kind of coverage to purchase; and second, if insurance premiums and co-pays depend on health behavior, such as smoking, exercise and diet, people will have incentives to improve their health, reducing the total costs of health care.

There is a growing body of research designed to measure the effects of policy alternatives with respect to individual responsibility and social cost sharing. One approach is to take advantage of cross-national data to measure the impacts of health insurance alternatives on patient behavior, population health and health disparities [7, 8]. That research shows that health outcomes in the U.S. have been decreasing in recent decades relative to other developed countries, who all practice universal cost sharing. However, given that many other nation-level variables co-vary with insurance policy [7, 8], it has been difficult to determine whether differences in health insurance policies are responsible for the trend and to pinpoint the causes of the decreasing relative status of the US.

Another approach is to take advantage of ‘natural experiments’ associated with policy changes, such the Oregon and other Medicaid Expansions [9–11], the SSDI Accelerated Benefits Demonstration Project [12], RomneyCare [13] and the Affordable Care Act (ACA)–ObamaCare [14] These studies confirm that health care usage increases in response to more generous insurance benefits. With the exception of three more recent studies they have been less conclusive, however, about the health benefits from the additional spending and services. There is evidence that self-reported health increases with increased health insurance coverage in Oregon [15] and with RomneyCare in Massachusetts [13], but effects on objective measures of health were not detected (see also [12]) with respect to mortality following the ACA. The assessment of long term impacts is limited by the fact that all are relatively recent reforms, in comparison to the decades over which chronic diseases develop. More recently, Miller et al. [16], Borgschulte and Vogler [17], and Goldin et al. [18] all find that the ACA reduced mortality.

There is also a rich literature of field experiments offering financial incentives for health promoting behavior. Giné et al. [19] show that financial incentives can promote smoking cessation. Volpp et al [20] show that incentives can induce subjects to lose weight in the short term, but in the long-term, post-incentive period the weight is regained. Incentives can also be used to increase gym attendance and exercise. Again, it is not clear that healthy behaviors are sustained in the post-incentive period. See [21, 22] for systematic reviews. In general, this research shows financial incentives influence people to adapt healthier behaviors. However, given their short time frames, such experiments are limited in their ability to measure the effects of interventions on health outcomes and cost.

These bodies of research illustrate the methodological challenges in assessing the effects of policy alternatives with respect to population health and total health care costs. The vast majority of healthcare costs and disease burdens are associated with important risk factors directly or indirectly behavioral, such as an obesogenic diet, lack of physical activity, smoking and drug use. In addition, the effects of behavior accumulate over many decades, suggesting that full policy impacts may accumulate slowly over time. Another challenge is that people can prepare for, and prevent, disease events along several different fronts. Examples are saving money, purchasing health insurance, changing habitual behavior, and engaging in preventative surveillance and treatment. Since different policies may affect the value of investing in those fronts, assessing total impacts will require an understanding of potential substitutions and complements between policies and individual behavior. Because of these challenges, there is very little causal evidence regarding the impacts of healthcare policy alternatives, despite the very precise statistics on costs and morbidity and the vigor of the policy debate.

Laboratory experiments can be important guides and complements to field experiments which are orders of magnitude costlier, riskier, and more time consuming. The laboratory offers many distinct advantages: the possibility to explore outside the realm of current feasibility, and the possibility to examine changes at the societal level, avoiding potential biases caused by ignoring counterfactuals due to selective participation and defection. Most importantly, the laboratory allows clear identification of moral hazard and adverse selection because the parameters of the environment are perfectly known to the experimenter, and measurements of all outcomes are precise and never obscured by difficult-to-discover costs or self-reported values. Finally, if a particular experimental treatment has negative consequences, they are directly manifested by diminished cash payoffs to participants rather than physical consequence in the real world. Salient results are guaranteed if participants simply prefer more reward to less [23]. Laboratory controlled decision-making experiments have proven to be a valid way to reduce complicated systems to essential operational features that can be used to test the impacts of rule-making policies before much costlier real-world implementation is attempted. For example, experimental electricity markets [24] have examined and improved the operational principles of the annual $14.5 trillion dollar energy industry (8.3% GDP), in which a tiny 1% improvement in efficiency is worth billions! Energy generation and distribution is fraught with complex non-linear dynamics and stochastic uncertainties, and it was highly regulated and long thought to be unamenable to incentive-based management. The fact that results from laboratory experiments with student subjects had external validity and were informative about about potential policy reforms in these complex markets, gives promise that controlled lab experiments can also provide insight into how policy alternatives might influence health, healthcare and insurance decisions.

This paper introduces a new, complementary experimental economic approach to address those challenges in examining the effects of health insurance policy alternatives on behavior and health outcomes over the life course. This experiment compares two types of premium systems for health insurance, one based on individual responsibility and the other on group-level cost and risk sharing.

Our experimental design tests predictions as to how cost sharing in health insurance premiums impact the following outcomes: 1) decisions about whether to purchase insurance; 2) behaviors that prevent or increase the risk of adverse health events; 3) overall health levels; 4) longevity; 5) savings; 6) overall welfare and 7) disparities in health and welfare due to income and age.

The paper proceeds as follows. The next section presents detailed descriptions of the the experimental environment and research design, the theoretical model motivating the experiment, and theoretical results which show how the optimal time paths of consumption and investments in health should vary as a function of the insurance and income treatments, from which we make predictions of subject behavior. Section three presents the empirical results and analyzes the treatment effects on subject behavior. The discussion section presents a summary of our main findings and discusses some weaknesses in the current design and appropriate directions for future research. The conclusion addresses how our approach complements existing approaches.

## Methods

### Model intuition

Our test environment, based on the Grossman [25] lifecycle model of health investment, is designed to model the multiple health-related decisions people make in their daily lives and the different ways people can prepare for and ameliorate disease events. Subjects live multi-period lives and have a stock of health that naturally deteriorates each period, and declines further if the subject suffers a stochastic illness event [26], which we call a health ‘shock.’ Each period, subjects receive an income (an allocation of virtual currency) that depends positively on their income class and their individual health [27] Subjects must then decide on how to allocate their income to current consumption versus health-related investments and savings. Health-related investments include the purchase of health insurance, the purchase of health recovery from deterioration and shocks, and expenditures on physical condition, which we call resilience, that lowers the size of potential future shocks. The ability to invest in resilience is designed to capture preventative behaviors such as exercise, diet and abstention from smoking, drugs, etc. whereas health recovery investment is meant to model services from medical providers, pharmaceuticals, etc. The probability of health ‘shocks’ increases during the second half of life to capture the effects of aging. Insurance pays for health recovery from shocks but does not compensate for natural deterioration.

### Treatments

The first treatment is actuarially-based insurance, in which the cost of each individual subject’s premium depends on that subject’s current expected health care costs to the insurer, which, in turn, depends on that subject’s age, health state, and health-related behavior. The actuarial plan embodies the concept of individual responsibility in a competitive insurance marketplace. Each subject chooses whether or not to purchase insurance given her personal premium. To alleviate the costs of premiums and continuing health degradation, subjects can invest in resilience, save to buffer against health shocks, and directly invest in improving current health. The actuarial plan smooths the lumpy costs of health shocks and provides incentives for individuals to improve their health. However, it may generate significant inequality in health outcomes, due to variable premium costs associated with individual conditions.

The second treatment is employer-based insurance, resembling the plans most Americans have. Premiums are largely subsidized by the employer (funded through a reduction in wages), and there is social cost sharing: all employees have no choice but to contribute to the employer’s costs through universal proportionate wage reductions. The premium the employer must pay the insurer is determined by the average expected costs of all employees who choose the plan. The proportion of the premium paid directly by the employee is fixed, regardless of age, income, health state or behavior. The employer treatment and its incentives differ significantly from the actuarial plan. First, since premiums do not depend on resilience, there is less incentive to invest in prevention (often referred to as, *ex-ante* ‘moral hazard’). (*ex ante* moral hazard concerns behavior that proceeds the insured event, and distinct from *ex post* moral hazard which concern behavior post event, such as seeking greater treatment than one would had they been uninsured.) Second, people with greater risk (the ‘old’) would have a greater incentive to purchase the insurance since their expected health costs would be greater (often referred to as, ‘adverse selection’). However, this plan is expected to generate less inequality in health outcomes due to social cost sharing.

Subjects were assigned to one of four experimental treatments groups in a 2x2 experimental design. One treatment dimension was the actuarial versus employer insurance plan, and the other was high versus low income. In addition, all subjects face a young and an old period during each life with the latter characterized by higher health risks (probability of shocks), so our statistical analysis is necessarily 2x2x2.

### Model

This subsection presents the general model employed in our experimental design. We build upon Grossman [25] where resources are limited and can be allocated between enjoying life and ensuring that it continues. Our subjects live for a maximum of *T* periods. They each have two state variables: health *H*_*t*_ and savings *S*_*t*_. Each period, health degrades by a fixed amount *δ*, and is improved through an investment of $I_{t}^{H}$ into health. Increasing health allows a subject to become more productive, earn greater income and spend less time attending to ailments; thus, income available to be spent by the subject during each period of the experiment is dependent on health. Any income not invested on health or current consumption is deposited into a savings account available for future consumption or investments in health. Subjects derive utility (which we refer to as ‘joy’ in the subject interface) from current investments in joy $I_{t}^{J}$. Joy is a concave function of dollars invested in consumption, moderated by health, as being healthy allows one to enjoy life more. Joy is our primary outcome and cumulative joy earned determines subjects payouts.

In addition to the standard elements of the Grossman model, we add the prospect of stochastic negative health shocks, and two additional investments, resilience $I_{t}^{R}$ and insurance $I_{t}^{I}$. Should a shock occur that period, resilience investment reduces the magnitude of the shock, while insurance, if purchased, will pay to recover any health lost due to the shock.

Listed below is the event timeline faced by subjects in our model. Each period, *t*, is subdivided into the following four stages:

1. Investments $\vec{I_{t}}=\{I_{t}^{H},I_{t}^{J},I_{t}^{R},I_{t}^{I}\}$ withdrawn from income and savings.
2. Intermediate health *H*_*t*′_ is determined for the current period considering health at the end of last period, health degradation *δ*, health improvement $R(H_{t},I_{t}^{H})$, and whether a stochastic health shock $\Delta_{t}\left(I_{t}^{R}\right)$ is realized.
3. Joy $J_{t}\left(I_{t}^{J},H_{t^{\prime }}\right)$ and period income *M*(*H*_*t*′_) are computed based on investments and intermediate health.
4. $\Delta_{t}\left(I_{t}^{R}\right)$ health is recovered if insurance was purchased and a health shock occurred, yielding a final health *H*_*t*+1_.

**Fig 1:**
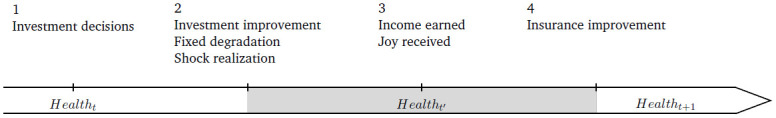
Sequence of events within a period.

Fig 1 shows the sequence of events each period and when health is at level *H*_*t*_ versus *H*_*t*′_. We included the mid-period health experienced *H*_*t*′_ so that subjects incurred a penalty from a shock even if insurance was purchased in order to capture the real-life utility and income costs of health shocks outside of medical bills. Placing insurance recovery at the end of the period is equivalent to a cash payout that is earmarked towards next period’s health, $I_{t+1}^{H}$, allowing for direct comparisons between insurance purchase and self-insurance through savings. (The health improvement function is additive with investments so that if *I* is the cash equivalent insurance payout, then $R\left(H_{t^{\prime }},I\right)+R\left(H_{t+1},I_{t+1}^{H}\right)=R\left(H_{t^{\prime }},I+I_{t+1}^{H}\right)$)

The equations that govern the individual’s welfare maximization problem are:

Maximize:

$$\begin{array}{cc} \sum_{t=1,T}J\left(I_{t}^{J},H_{t^{\prime }}\right) & \left(\text{Lifetime}\;\text{Joy}\right) \end{array}$$

Subject to:

$$\begin{array}{cc} H_{t^{\prime }}=H_{t}-\delta +R\left(H_{t},I_{t}^{H}\right)-x_{t}\Delta \left(I_{t}^{R}\right) & \left(\text{Health}\;\text{Experienced}\right)\\ H_{t+1}=H_{t^{\prime }}+D_{t}^{I}x_{t}\Delta \left(I_{t}^{R}\right) & \left(\text{Health}\;\text{at}\;\text{start}\;\text{of}\;\text{next}\;\text{period}\right)\\ S_{t}+\sum_{k}I_{t}^{k}=S_{t-1}+M\left(H_{{\left(t-1\right)}^{\prime }}\right) & \left(\text{Budget}\;\text{constraint}\right) \end{array}$$

Where:

$J(I_{t}^{J},H_{t^{\prime }})$ is the joy realized in period t.

*x*_*t*_ is the realization of the shock degradation random variable *X*_*t*_ ∼ Ber(*μ*_*t*_), where *μ*_*t*_ is a fixed probability of a shock depending only on age.

$I_{t}^{I}=D_{t}^{I}\rho_{t}(H_{t^{\prime }},I_{t}^{R},\mu_{t})$, with $D_{t}^{I}\in \{0,1\}$ being the binary decision whether to purchase insurance, and $\rho_{t}(H_{t^{\prime }},I_{t}^{R},\mu_{t})$ being the insurance premium that may depend on health, shock size and shock probability.

#### Functional forms and parameters

Below we delineate the specific functional forms we employed in the experiment in order to implement the general model discussed above. We begin with the functional forms of the joy, health regeneration, resilience and income functions.

$$J_{t}\left(I_{t}^{J},H_{t^{\prime }}\right)=\frac{I_{t}^{J}}{\left(\gamma +I_{t}^{J}\right)}H_{t^{\prime }}$$ (1)

$$R(I_{t}^{H},H_{t})=\frac{e^{\alpha I_{t}^{H}}}{e^{\alpha I_{t}^{H}}+\left(1-H_{t}\right)/H_{t}}-H_{t}$$ (2)

$$\Delta (I_{t}^{R})=\Phi e^{-rI_{t}^{R}}$$ (3)

$$M(H_{t^{\prime }})=H_{t^{\prime }}\Omega^{c}$$ (4)

The joy function, Eq 1, has diminishing marginal returns to consumption, *I*^*J*^ and moderates joy realized by health experienced. The consumption term is asymptotic so that like the health term it has an upper bound. The health regeneration function, Eq 2, is S-shaped logistic. Improving health is least expensive at the middle of it’s range, and progressively more expensive toward either end of the range. It is more expensive when health was high to model diminishing marginal returns, and more expensive when health was low to model the complication of treating co-morbid health issues. This specification also ensures that cost of increasing health remains the same no matter how many periods the subject takes to do so. The shock moderation function, Eq 3, imposed diminishing returns to investments in resilience, *I*^*R*^, the ability to moderate the magnitude of shocks. The income function, Eq 4, was linear and increasing in health experienced. Additionally, income depended on the subject’s base income type, low or high.

In the experimental environment that is used to implement this model, the subject lives at most 32 periods, but can die earlier. Health is restricted to values from 100 (perfect health) to 0 (death). Life starts with health at 70 (*H*_0_ = 70) and depreciates by 8 (*δ* = 8) each period with certainty. In addition, health declines further if there is a shock in a given period. The shock probability is 3/12 in the first half of life (*μ*_*t*_ = .25 for *t* ≤ 16), and 5/12 in the second half (*μ*_*t*_ = .4167 for *t* > 16). The unmitigated shock was set to 25 health points (Φ = 25), but could be reduced to as low as 12 through investment in resilience *I*^*R*^. We use Eq 3, but impose a maximum investment per period and limit it to take on integer values. The shocks are pseudo-random. We use 12 pre-generated shock sequences that rule out low probability sequences (< 10 and > 12 shocks/lifetime). Each subject receives a different shock sequence from life to life and and from other members of a cohort with whom (s)he is allowed to chat.

To explore the heterogeneous impacts of insurance plans we create two types of variation in the population. Subjects are either high- or low-income, with their type constant equal to the most they could earn in any given period. Realized income for both types is their type constant multiplied by their health. The constant Ω^*c*^ for the high-income type was 200, 1.25 times greater than the one for the low-income type, 160. While we refer to the income types as high and low, both types had sufficient income to purchase insurance and might be better described as high and middle income. For all levels of health below 35, the subjects received the minimum income, the same amount as if they had a health of 35. For both income types we increase the probability of health shocks during the second half of life when the subjects become old, creating an addition level of treatment variation.

Under the ‘employer’ insurance plan, the employer paid 75% of the insurance premium cost, which subsidy was fully funded through wage reductions that were universally imposed on all employees, regardless of whether the individual chose to purchase the insurance. All employees were offered the opportunity to purchase insurance at a fixed cost that was approximately 25% of the real cost of insuring the average person in the employer’s pool. The constant deduction rate across incomes (18%) meant that the system transferred contributions from high- to low-income subjects, while the constant premium cost regardless of age transferred contributions from young to old subjects. Calculations of exact amounts are in S2 Table in S1 File. Our environment is mathematically equivalent to an overlapping generations’ model. (Though we could have transferred subsidies from young to old within a period, we avoided the logistical complications of having subjects of varying ages participating simultaneously by pre-computing the actuarially accurate transfers to that needed to be implemented.)

Under the actuarial plan, greater individual responsibility requires a complex and delicate dynamic mix of savings, investments in resilience and health, and timely purchase of insurance. Moreover, because they are unconstrained by an imposed employer tax and subsidy, individuals under the actuarial plan have the potential to obtain greater total welfare. Under the employer plan the discounted insurance premium funded by the salary tax predicts the universal purchase of insurance, but reduced individual savings. In addition, the flat premium for all individuals under the employer plan predicts moral hazard leading to reduced investment in resilience to shocks.

Under both insurance treatments, investment in resilience and the purchase of insurance are partial substitutes for one another. Purchasing insurance negates the benefits of resilience in future periods since any insured shock is immediately recovered from. However, resilience mitigates a shock’s immediate impact on joy and income—insurance does not provide this benefit. Conversely, investing in resilience reduces the value of the insurance. Under the actuarial treatment insurance and resilience are also complements. Because insurance premiums are calculated to be actuarially fair, conditional on purchasing insurance, the marginal cost of resilience is implicitly reduced as it also results in lower insurance premiums.

#### Dynamic programming based predictions

This environment is very complex and the optimal path through life is not clear. The combination of a finite lifetime of uncertain duration, stochastic health shocks, and dynamic interdependence across 5 possible investments, some with nonlinear profiles, make a closed form solution intractable. Yet we regard this life course navigation task as less difficult than the behavioral enterprise faced by human subjects needing to evaluate what, amongst the millions of investment/consumption decisions they will need to make during their lives and calculating today’s marginal lifetime value of; doing 30 minutes more of exercise or not drinking a soda. Furthermore, as a surrogate for inter-generational and media based wisdom, we gave our subjects practice lives and group chat, to aide them in navigating their decisions.

Lacking a closed form solution, we encoded a computerized stochastic dynamic program (DP) that finds the optimal investment path for a subject by automatically readjusting to maximize expected remaining lifetime joy given any possible shock sequence that might be encountered.

**Fig 2:**
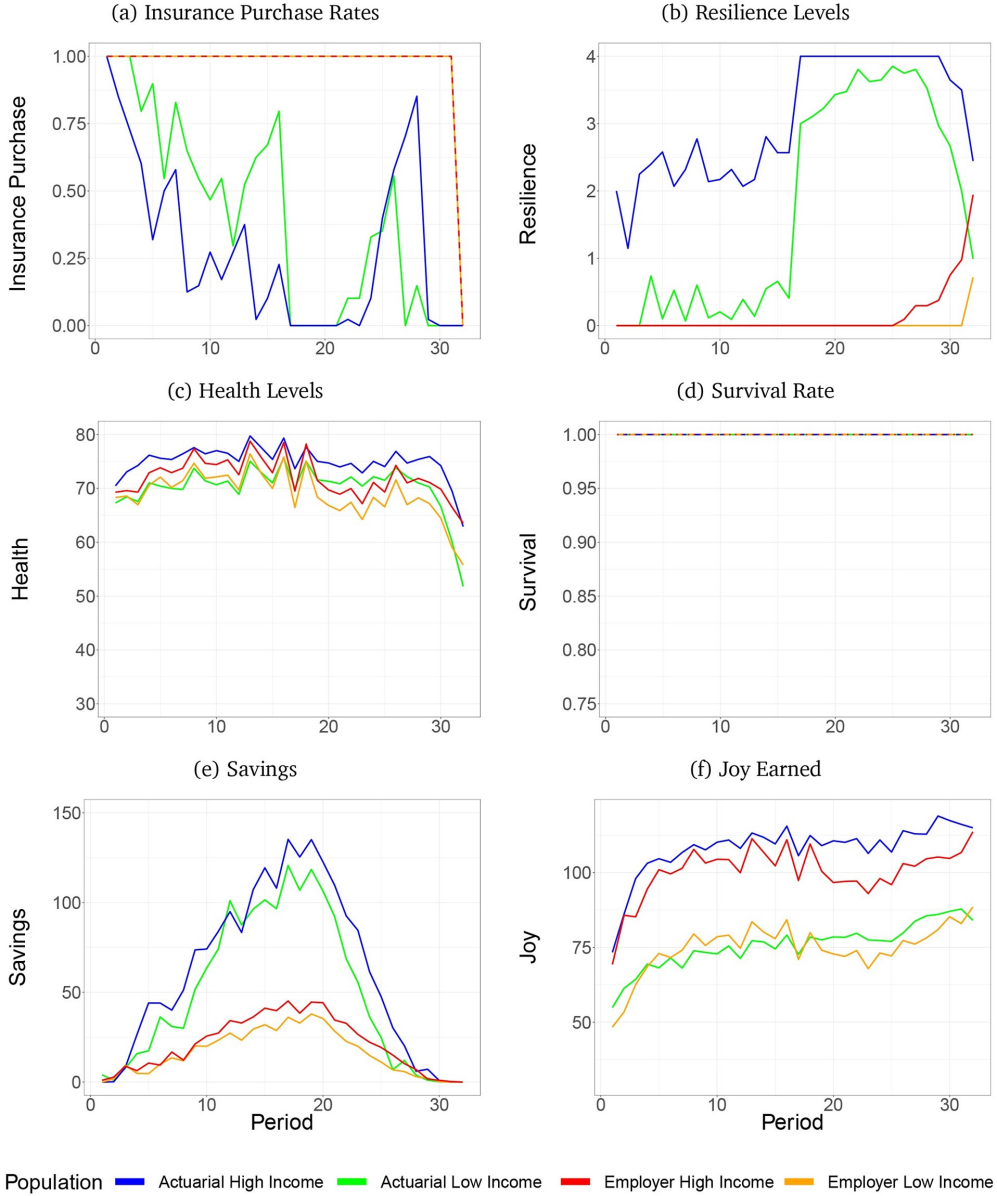
Results from dynamic program. Includes panels: (a) Insurance purchase rates, (b) Resilience levels, (c) Health levels, (d) Survival rate, (e) Savings, (f) Joy earned.

Fig 2 shows the mean optimal results generated by the dynamic program for the four treatment groups. Red and orange lines plot the mean joy maximizing strategies in the employer treatment for high and low-income subjects, and blue and green do the same for the actuarial treatment. (The somewhat jagged results occur because subjects are limited to discrete levels of investment, which can result in sudden switches in optimal investment levels between compliments or substitutes especially when confronted with a change in shock probability.) The results derived from the mean investment choices made by optimizing agents reveal that optimal strategies clearly depend on both insurance plan and income, and change dynamically through life, and as shocks are encountered. We do not expect our human subjects to navigate the optimal expected investment paths revealed in the DP figures, however, the mean optimal paths suggest the qualitative properties of the dynamic paths that optimizing subjects responding to incentives would follow. We employ the DP results to generate the theoretical predictions about the effects of the two insurance treatments on subject behavior and how those interact treatments interact with the income and life-stage treatments.

*Insurance purchase*. Panel a plots the proportion of the 12 optimizing agents that purchase insurance in each period. The theoretical results generate two predictions with respect to insurance treatment effects on the decision to purchase insurance:

**Prediction 1**: In the actuarial treatment, lower income subjects will purchase insurance at higher rates than higher income subjects, (optimizing actuarial agents tended to invest in resilience and self insure, thus did not purchase insurance unless they encountered shock realizations that depleted the savings required for self insurance. This will occur more often for low-income subjects) but there will be no income effect in the employer treatment where all subjects are always predicted to purchase insurance.
**Prediction 2**: Subjects offered the actuarial plan will purchase insurance about half the time, and will be more likely to purchase insurance when they are very young with little savings or very old; the short time horizon makes insurance a better value than resilience.

*Resilience*. Panel b plots mean resilience level to shocks throughout life.

**Prediction 3**: Within the actuarial treatment, subjects will increase their investments in resilience in the latter half of life in response to the increase in the probability of a shock.
**Prediction 4**: Due to moral hazard in the employer treatment, subjects in this treatment will not invest in resilience until near the very end of life. This is in sharp contrast to the actuarial treatment, in which subjects invest in resilience at near maximal values when old.

*Health*. Panel c shows health experienced by optimizing agents.

**Prediction 5**: Subjects in both treatments will increase health from the endowed starting level and maintain a target level of health until the final 4-5 periods of life.
**Prediction 6**: There will be minimal differences in health levels across age, income or insurance treatments.

*Survival*. Panel d shows the proportion of optimizing agents alive in each period.

**Prediction 7**: In all treatments, subjects will survive to the final period of life.

*Savings*. Panel e reveals that optimizing agents will transfer money from earlier periods to later periods to smooth consumption and maintain health level, despite the increased shock prevalence in the second half. The employer plan’s pricing reduces the need for a subject to save.

**Prediction 8**: In both insurance treatments, savings will increase in the first half of life and then decrease in the second half of life.
**Prediction 9**: Savings will be about four times higher in the actuarial than in the employer treatment, exceeding 100 at midlife.

*Joy*. Panel f shows optimizing agents largely smooth joy across life. Joy investments are low in the beginning of life when the priority is increasing health and future income. Conversely, at the end life joy investment is increased:

**Prediction 10**: For high income subjects, joy obtained will be higher in the actuarial treatment than in the employer treatment throughout life.
**Prediction 11**: For low income subjects, joy obtained in the employer treatment will be higher during the first half of life, but lower during the second half of life.
**Prediction 12**: For high income subjects, aggregated lifetime joy will be substantially higher in the actuarial treatment than in the employer treatment. For low income subjects, aggregated lifetime joy will be marginally higher in the actuarial treatment than in the employer treatment.

### Experimental interface

**Fig 3:**
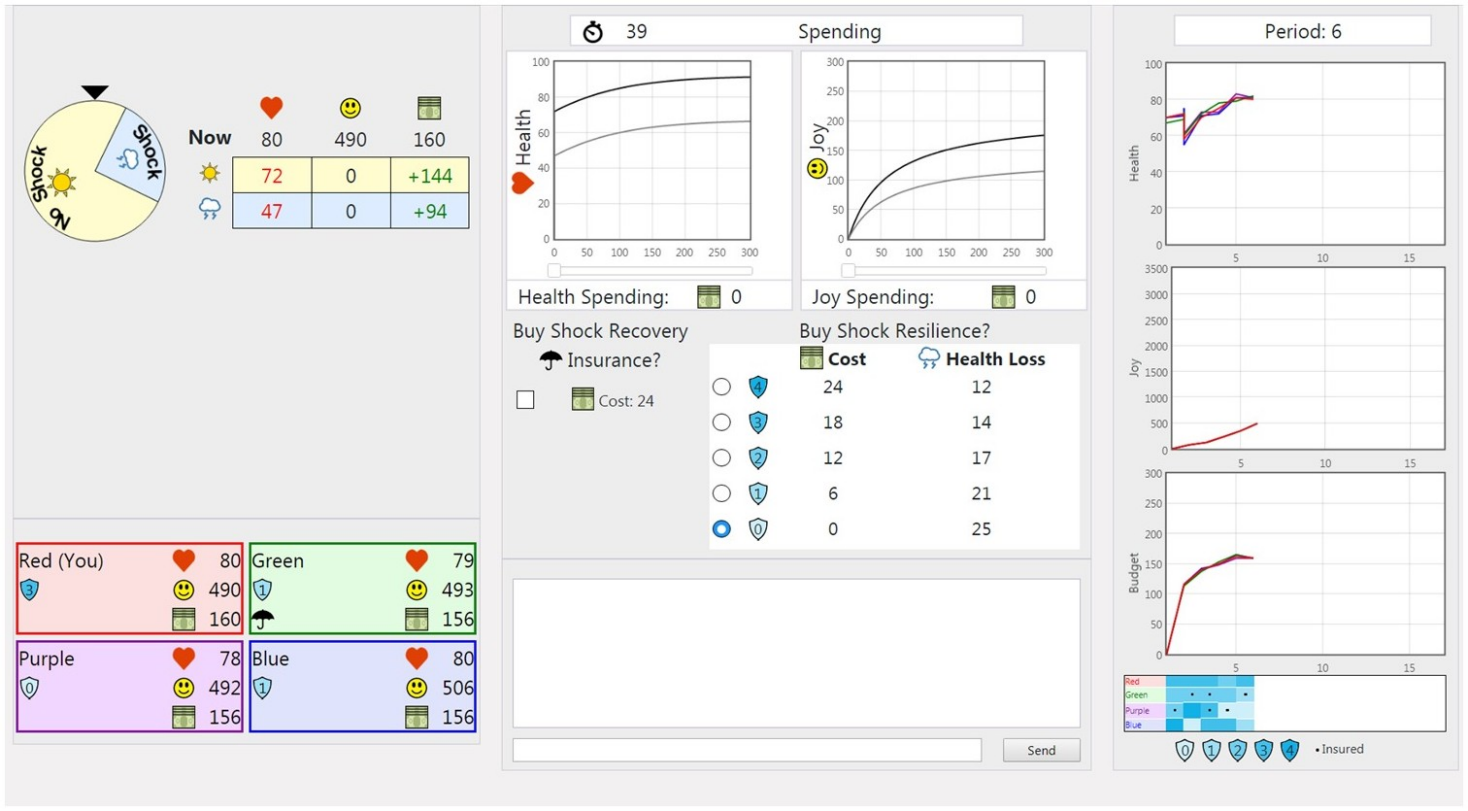
Subject interface.

Fig 3 shows the experimental interface seen and manipulated by the subjects. The heart icon represents health, the happy face denotes joy, and the money icon cash. The row above of the small table shows the present values of health and accumulated joy, and the income the subject will receive this period. The two rows in the table show the resulting health, marginal joy earned, and balance of the savings account at the beginning of next period given a shock is not or is encountered. Money can be allocated to health or joy by moving the respective sliders below the graphs in the top center. The graphs allow the subject to see the diminishing marginal returns of both investments. Subjects make insurance and resilience decisions by checking boxes below the graphs. Remaining budget and potential outcomes update dynamically with each decision. Any unallocated money is automatically saved, and added to income next period. (Saving was possible for any number of periods, but not across lives. Any savings held at the end of a life were lost.) Subjects were paid a fixed show up fee, and the sum of joy received across all periods and lives multiplied by fixed conversation rate; they were induced to maximize utility.

During the shock stage of the period following the investment stage, the shock wheel in the upper left corner spins. If the pointer lands on the darker section with the storm cloud icon, the subject receives a shock. The darker shock section of the wheel is proportionately larger during the second stage of life indicating the increased probability of a shock. The lower lines in the investment graphs indicate diminished values when a shock occurs. The outcome stage of the period is when the subjects receive both joy and income. It was temporally separated to make it clear that both were based on the subjects’ health, including reductions from any shock received during that period. In the final stage, if the subject had bought insurance that period, health was restored to its pre-shock level. If the subject was uninsured or self-insuring through savings, they could only pay to restore their health in the first stage of the following period.

Subjects are organized into groups of four individuals with the same income type. The right side of the screen shows the group history for the present life. There are graphs for health, cumulative joy, and budget. Each graph has a colored line for each group member that corresponds with the member’s title color and label. The bottom right corner displays each group member’s resilience history in a heat map style table, with superimposed dots indicating insurance purchase decisions. The numbers in the shields below the table indicate resilience levels corresponding to color density. The colored coded tables at the bottom left reveal the current status of each group member. The umbrella icons indicate being currently insured.

Subjects could choose whether to chat with other group members through text. The bottom center is the chat section. Chat is available at all times including in pauses between lives. Even though subjects can chat, subject payouts from the experiment depend solely on individual performance. There is no financial reward to sharing information, nor is there any incentivized competition giving reason to withhold information.

After thorough on screen and audio instructions, subjects participate in three practice lives of 4, 16 and 16 periods, before engaging in the two longer 32 period lives that we analyze. Subjects are paid for all periods of all lives. A subject who dies (i.e. Health = 0) in any particular life must wait until all the other subjects in the experimental session complete that life, but can chat with other group members and observe all their actions.

### Procedures

We ran 12 experimental sessions at the Economic Science Institute (ESI) Lab at Chapman University between April 24^th^ and May 16^th^, 2018. Eight sessions had 24 subjects, two had 20, one had 16 and one had 12. (When there were fewer than 24 subjects, the number of subjects was a multiple of four so that all subjects had equal-sized groups. The subjects, who remained after the maximum number of groups of four was formed, were paid a show-up fee and allowed to depart.) Subjects were recruited via email from a database of subject volunteers ESI maintains. Sessions lasted 120 minutes. Average subject earnings were $36.79, including the $7 show-up fee. Table 1 shows the 2x2 treatment design and number of subjects in each cell. We analyze 64 periods (two 32-period lives) for each subject, yielding over 4,000 observations per cell.

**Table 1:** Subjects per treatment cell.

	Treatment	
	Actuarial	Employer
Low income	64	68
High income	64	64

Table 2 lists insurer details by treatment. The insurance premium charged in the Actuarial treatment was the expected cost of treating the shock plus 10% (to model administrative costs). The insurance premium was always $9 in the Employer treatment, however subjects in the Employer treatment received only 82% of the base income they would have received in the Actuarial treatment. The ‘employer’ uniformly decreased wages by 18% in order to fund the insurance subsidy it provided plus pay the insurer.

**Table 2:** Treatment details.

	Actuarial	Employer
Premium	1.1 * *E*(*Cost*_*it*_)	$9
Subject income	100%	82%
Withheld income	0	654
Premiums collected	442	259
Total revenue	442	913
Benefits paid	409	865
Insurer profit	$32	$48
Profit rate	7.3%	5.2%

Notes: Insurer revenue is defined as the sum of all premiums paid (in both treatments) plus all income reductions to subjects (in the employer treatment). Profit is defined as revenue minus the total amount paid to the subjects to recover from insured shocks. The profit rate is the profit divided by the revenue.^a^ Values reported are per subject lifetime. The actuarial treatment had a slightly lower profit rate (7.3%) than designed, because, by chance, the stochastic shock rate on the periods when subjects did not buy insurance was lower than the rate during periods when insurance was purchased. The insurer profit rate in the employer treatment (5.2%) was slightly lower than in the actuarial treatment, however, the revenue stream was larger due to much increased subscription, so overall the insurer made more profit under the employer plan ($48) than the actuarial plan ($36).

^*a*^ *Row*5 = *Row*3 + *Row*4; *Row*7 = *Row*6 − *Row*5; and *Row*8 = *Row*7/*Row*5.

The table demonstrates that the subsidies under the employer plan were completely funded, and no ‘external’ resources were required.

### Data analysis

Our regressions on insurance purchase and survival will use a panel data probit model:

$$\begin{array}{c} Y_{it}=\Pi \left(\phi_{i}+\tau_{i}+\nu_{i}+\varepsilon_{it}\right) \end{array}$$

where ***ϕ***_***i***_ is a vector of sub-population binary variables indicating the subject’s age within the experiment life {young, old} and income type {low, high}; ***τ***_***i***_ is vector of binary variables indicating belonging to a particular sub-population and being in the employer-based insurance treatment; *ν*_*i*_ is an individual error term; and *ε*_*it*_ is period and subject specific error term. Errors are clustered according to which shock sequences (there were only twelve) the subjects experienced.

Our regressions on resilience, health, savings and joy will use a panel data model:

$$\begin{array}{c} Y_{it}=\phi_{i}+\tau_{i}+\nu_{i}+\varepsilon_{it} \end{array}$$

where the variables have the same specifications, but there is no link function.

## Results

**Fig 4:**
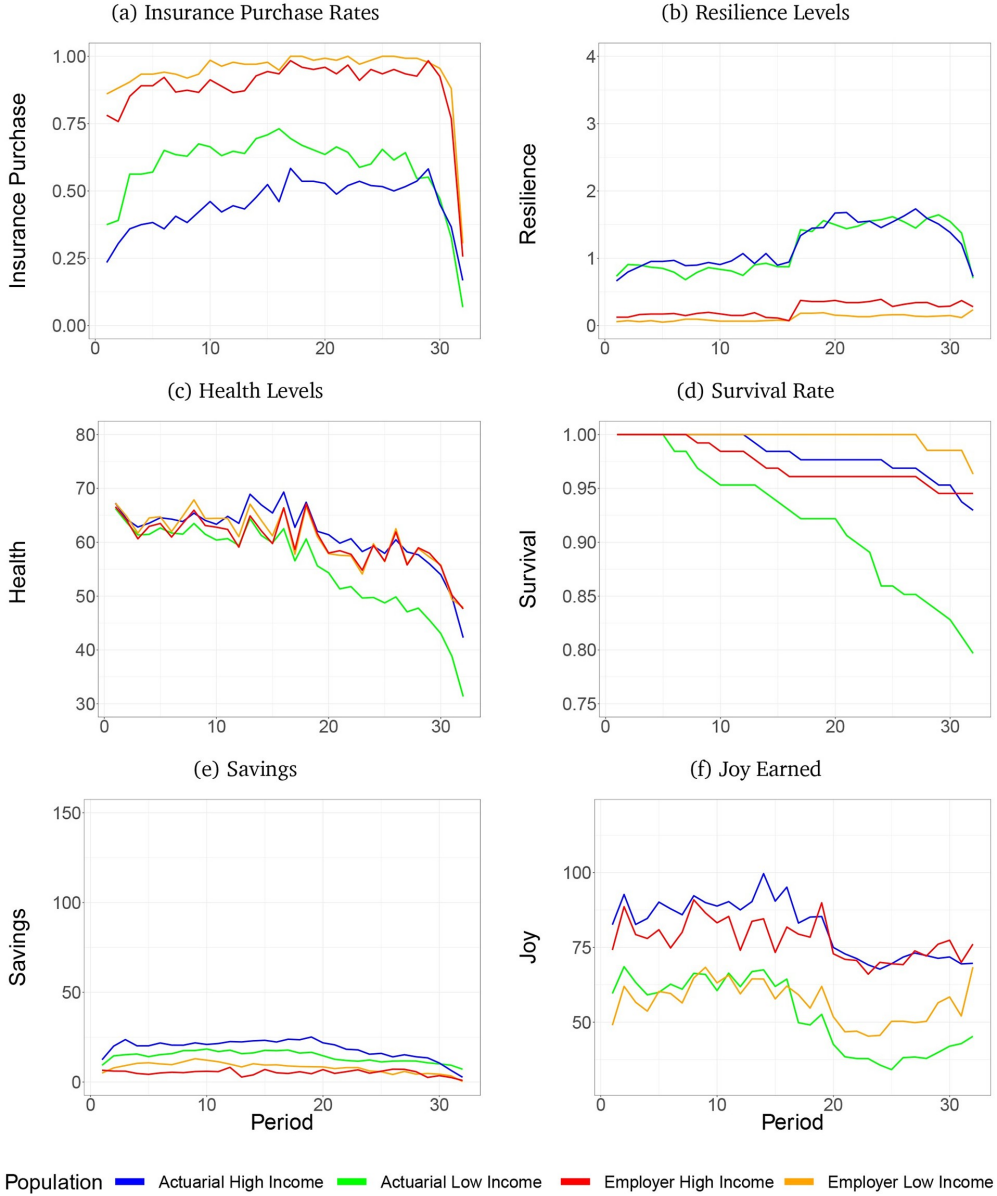
Results from subjects. Includes panels: (a) Insurance purchase rates, (b) Resilience levels, (c) Health levels, (d) Survival rate, (e) Savings, (f) Joy earned.

Fig 4 presents temporal plots of mean subject data by treatment. Red and orange lines plot behavior in the employer treatment for high and low-income subjects, and blue and green plot the actuarial treatment. (S1 Table of the S1 File provides the summary statistics for the subject data plotted.) We use the plots to render subject behavior in the environment before proceeding to regression analysis of the differences across populations and treatments.

### Insurance purchase

Panel a shows, that nearly all subjects of both income groups in the employer treatment purchased insurance every period except the last one or two. Under both insurance treatments, low-income subjects purchased insurance at greater rates than high-income subjects.

### Resilience

Subjects invested more in resilience in the actuarial than in the employer treatment, and investments in resilience increased in the second half of life when the shock rate increases (Panel b).

### Health

Subjects maintained relatively high levels of health throughout midlife (Panel c). However, in all treatments health tapers off sooner during the second half of life. This results in lowered incomes throughout life because and less joy realized during later periods of life. The effect is more pronounced for subjects in the actuarial treatment, especially low-income subjects.

### Survival

Panel d shows some subjects died due to under-investments in health and resilience, especially in the low-income actuarial treatment in which 20% died.

### Savings

Subjects in the actuarial treatment saved more than those in the employer treatment, and high-income subjects save more than low-income subjects (Panel e).

### Joy

As was achieved by the optimizing agents, Mean joy is higher in the actuarial than in the employer treatment for higher income subjects, especially during the first half of life (Panel f). However, in contrast, low-income subjects realized less joy in the actuarial than in the employer treatment, particularly during the second half of life.

### Regressions on treatment period differences

Table 3 reports regression results that provide statistical tests of the treatment differences illustrated by the results graphs. The six table columns labels (a-f) correspond respectively to the six panels in the result graphs. For all columns, estimates are reported for seven binary indicator variables. The first three variables represent the four sub-populations resulting from interacting age and income (low-income young is the omitted sub-population), so each variable provides an estimated impact of belonging to that sub-population relative to the omitted one. The remaining four variables indicate both belonging to a particular sub-population and being in the employer-insurance treatment, so they estimate specific effect (of employer-insurance) for each of the four sub-populations.

**Table 3:** Regression estimates for baseline differences and treatment effects.

VARIABLES	(a)	(b)	(c)	(d)	(e)	(f)
	Ins. purchase	Resilience	Health	Survival	Savings	Joy
Low income old	-0.0760***	0.532***	-13.14***		-1.542	-22.10***
	(0.0201)	(0.0886)	(1.669)		(3.169)	(2.788)
High income young	-0.150***	0.0991	3.132*		5.692**	25.93***
	(0.0364)	(0.0950)	(1.656)		(2.649)	(2.006)
High income old	-0.0852***	0.676***	-4.008	0.117**	1.373	10.15**
	(0.0262)	(0.0966)	(2.591)	(0.0527)	(2.881)	(4.136)
Emp * low young	0.372***	-0.692***	2.410		-5.645	-3.016
	(0.0362)	(0.0496)	(1.797)		(3.739)	(2.303)
Emp * low old	0.408***	-1.144***	8.401***	0.127***	-7.906***	11.62***
	(0.0340)	(0.0716)	(2.579)	(0.0244)	(2.897)	(2.743)
Emp * high young	0.414***	-0.711***	-2.103*		-15.61***	-8.239***
	(0.0293)	(0.0863)	(1.267)		(1.925)	(2.336)
Emp * high old	0.352***	-1.124***	-0.481	0.0224	-10.81***	0.252
	(0.0258)	(0.0847)	(2.138)	(0.0686)	(1.619)	(3.803)
Observations	16,640	16,640	16,640	455	16,640	16,640
Sequence controls	Yes	Yes	Yes	Yes	Yes	Yes
Log likelihood	-6139			-127.9		
Rho	0.513	0.467	0.461	0.0949	0.612	0.220
P-value		<.001	<.001		<.001	<.001
Number of subj. lives		520	520		520	520

Clustered (on Shock Sequence) standard errors in parentheses

*** p<0.01,

** p<0.05,

* p<0.1

The following results are summarized in Table 6.

#### Insurance purchase

Column (a) of Table 3 presents estimated marginal effects of a probit regression on insurance purchase. In the actuarial treatment, the low-income, young subjects purchase insurance at the highest rate, because they are most vulnerable–less able to afford resilience or to self insure through savings. The difference between the purchase rate and each of the other three sub-populations is statistically significant, as is reported in the first three rows.

**Result 1**: ★ As predicted, within the actuarial treatment, low-income subjects. (The estimate in row 2 is negative and the estimate in row 3 is less than row 1.)
**Result 2**: As predicted, in the employer treatment, subjects are (35 to 41%) more likely to purchase. (Rows 4 to 7 provide estimate effect for each type.)

#### Resilience

Column (b) of Table 3 reports coefficient estimates from a linear regression on resilience (ordered probit results are in the S3 Table in S1 File).

**Result 3**: As predicted, subjects in the actuarial treatment increase resilience during the second ‘old’ half of life (rows 1 and 3 are positive), but much less than predicted (the values are less than 1).
**Result 4**: Consistent with predicted moral hazard, resilience is lower in the employer treatment. (Rows 4 to 7 are all negative). ★ However, the difference is smaller than predicted.

#### Health

Column (c) of Table 3 reports coefficient estimates from a linear regression on health. Health is dramatically lower, over 13 points, for low-income old subjects. Health was maintained by all sub-populations at lower levels than counterpart optimizing agents.

**Result 5**: ★ Contrary to the DP prediction, subjects do not increase their health from initial levels. Subject health declines as predicted in the end of life, though to a smaller magnitude.
**Result 6**: ★ In the actuarial treatment, there are larger than predicted health disparities between high and low income subjects. For young subjects high income produces significantly higher health (row 2 is positive), and for old subjects high income produces dramatically higher health (row 1 is large, negative and statistically significant, while row 3 is not different than 0). The employer treatment largely diminishes these disparities for both low income (adding row 5 to row 1 is -4.74, only about a third of row 1) and high income subjects (the sum of rows 6 and 2 is nearly 0).

#### Survival

Column (d) of Table 3 reports probit estimates on whether the subject was alive at the end of the 32 period life, so there are no estimates for young subjects. Sixty-five subject lives were dropped from the regression because their innocuous shock sequences perfectly predicted survival.

**Result 7**: The survival rate across all subjects was 91% whereas no optimizing agent failed to survive.
  A) Under the actuarial treatment, being high-income increased a subject’s chance of survival by 11.7% (row 3).
  B) ★ Employer insurance closes the income survival gap (rows 3 and 5 are nearly equally) as fewer low-income subjects die prematurely, without significant impact on the survival of high income subjects (row 7 is near 0).

#### Savings

Column (e) of Table 3 shows regression estimates of how savings differed by sub-population. Only the high-income young had a statistically significant difference in savings. The employer treatment reduced savings for all four sub-populations, but the reduction for low-income young subjects was not statistically significant.

**Result 8**: ★ Contrary to the DP prediction, subjects in all treatments did not accumulate substantial savings throughout the first half of life in order to smooth health and consumption during the second half of life. Subjects saved for the first couple of periods, achieved some (relatively low) savings target, and then maintained that low level target until midlife, after which they slowly dispensed their savings.
**Result 9**: ★ This lack of savings for future use was especially dramatic in the actuarial treatment. While the regression coefficients are statistically significant, their magnitude is too small. Savings was only a fraction of what was required to smooth health and consumption.

#### Joy

Column (f) reports estimated differences in joy obtained within periods. Young subjects earn more than old, and high-income earn more than low-income.

**Result 10**: As predicted, for high-income subjects, the employer treatment reduces joy obtained when they are young (row 6). ★ However, contrary to the DP prediction, there is no impact on joy obtained when subjects are old (row 7).
**Result 11**: ★ Contrary to the DP prediction, low income subjects in the employer treatment obtained joy equal to those in actuarial early in life, and then surpassed them later in life (row 5).

### Insurance treatment effect on lifetime joy

Table 4 provides regression estimates of the cumulative effect of employer insurance on lifetime Joy over the 32 periods of life by income category.

**Table 4:** Coefficient estimates from regressing cumulative joy on insurance type.

VARIABLES	(1)	(2)	(3)
	Low income	High income	All
Employer	137.7*	-127.8	-5.127
	(72.75)	(90.03)	(76.43)
Observations	264	256	520
# of Subjects	132	128	260

Robust standard errors in parentheses

*** p<0.01,

** p<0.05,

* p<0.1

**Result 12**: ★ Contrary to the DP prediction, the employer treatment reduces lifetime Joy disparity (column 1 is positive), without a statistically significant impact on aggregate population Joy (column 3 is not statistically significant).

Contrary to the DP prediction that welfare will be higher in the actuarial plan because subjects were unconstrained by an imposed employer subsidy/wage contribution, welfare was actually higher in the employer plan. Table 5 investigates this inconsistency, comparing mean joy subjects earned in the Actuarial and Employer Treatments to mean joy earned by optimizing agents (the DP means). It confirms significant interaction between income and insurance treatment in obtaining joy. High-income subjects obtained more joy in the actuarial than in the employer treatment, but contrary to the optimizing agents, low-income subjects fared absolutely better in the employer than in the actuarial treatment. As such, differences in obtained joy between low and high-income subjects were significantly greater in the actuarial than in the employer treatment, with low-income subjects obtaining 73% of the joy high-income subjects obtained in the employer treatment, but only 64% in the actuarial treatment. Similarly, if we compare, efficiency, subjects’ performance relative to the mean of the optimizing agents, low-income actuarial subjects are the clear outliers, obtaining only 69% of their optimizing counterparts’ joy, whereas the other three groups achieve 76% ± 1%.

**Table 5:** Mean joy obtained.

	Low income			High income			Low/High	
	Subject	Optimal	Efficiency	Subject	Optimal	Efficiency	Subject	Optimal
Employer	1816	2391	0.76	2482	3213	0.77	0.73	0.74
Actuarial	1679	2423	0.69	2609	3462	0.75	0.64	0.7
Emp/Act	1.08	0.99		0.95	0.93			

## Discussion

The Results of the previous section are succinctly summarized in the Table 6 below.

**Table 6:** Summary of predictions and results.

Outcome	#	Prediction	Result as predicted?
Insurance purchase	1	Actuarial, lower income subjects purchase at higher rates than higher income subjects, but there’s no income effect in the employer treatment where all are predicted to purchase.	★ Yes.
	2	Actuarial subjects purchase 1/2 the time, and are more likely to purchase when very young with low savings or very old, when the short horizon makes insurance better than resilience.	Yes, but to a lesser extent.
Resilience	3	Actuarial subjects increase investment later in life in response to increased probability of shock.	Yes, but to a lesser extent.
	4	Due to moral hazard employer subjects do not invest in resilience until the very end of life. In sharp contrast, actuarial subjects invest in resilience at near maximal values as they age.	★ Yes, but the difference is smaller than predicted.
Health	5	All subjects increase health from the endowed starting level to maintain a target level.	★ No.
		All precipitously decrease in final 5 periods.	Yes, but to a lesser extent.
	6	There are minimal differences in health levels across age, income or insurance treatments.	★ No, under actuarial, there are larger health disparities between high and low income subjects. High income produces significantly higher health for young, and dramatically higher health for old. The employer treatment largely diminishes these disparities across income level.
Survival	7	In all treatments, subjects will survive to the final period of life.	No, under actuarial, higher income increased subject’s chance of survival by 11.7%. ★ Employer insurance closes the income survival gap.
Savings	8	Savings will increase in the first half of life and then decrease in the second half.	★ No, subjects saved a few periods, maintained until midlife, then slowly dispensed.
	9	Savings is four times higher under actuarial than employer, exceeding 100 at midlife.	★ No, lack of savings for future use was especially dramatic in the actuarial treatment.
Joy	10	For higher income subjects, joy obtained is higher in the actuarial treatment than in the employer treatment throughout life.	The employer treatment reduces joy obtained by higher income young subjects; ★ but, it does not reduce joy obtained by older subjects.
	11	Joy obtained by lower income employer subjects is higher during the first half of life, but lower during the second half.	★ No, lower income employer subjects obtained joy equal to actuarial subjects early in life, and surpassed them later in life.
Lifetime joy	12	For higher income subjects, aggregate lifetime joy is substantially higher in the actuarial treatment than in the employer treatment.	No.
		For lower income subjects, aggregate lifetime joy is marginally higher in the actuarial treatment than in the employer treatment.	No.

Taken together, these results show that subjects were sensitive to the differential incentives in the actuarial and employer treatments. In the employer treatment, subsidies did provide an incentive to purchase more insurance (Result 1), and its fixed cost, regardless of subject behavior, reduced investments in resilience, supporting the moral hazard prediction (Result 4). However, subjects in both treatments under-invested in the future in favor of current consumption. In aggregate, subjects spent 10.7% more of their budget on current consumption than was optimal, as determined by the DP optimizing strategy, given the period, present budget and health. This is evident in the underinvestment in savings (Result 9) and in health early in life by subjects in both the employer and actuarial treatments (Result 5). Moreover, in the actuarial treatment, subjects also underinvested in resilience (Result 4), undervaluing the marginal effects of resilience in reducing the size of a health shock, and failing to respond to incentives in insurance pricing.

The under-investment in the future and over-investment in current consumption had greater impacts on total joy obtained in the actuarial than in the employer treatment. Investments in savings and resilience are more critical to obtaining joy in the actuarial treatment: savings, because insurance costs increase in the second half of life, when the shock probability becomes higher; and resilience because insurance premiums vary negatively with investments in resilience. The flat lifetime rate for insurance in the employer treatment both obviates the need for significant savings and provides little incentive for investments in resilience.

**Fig 5:**
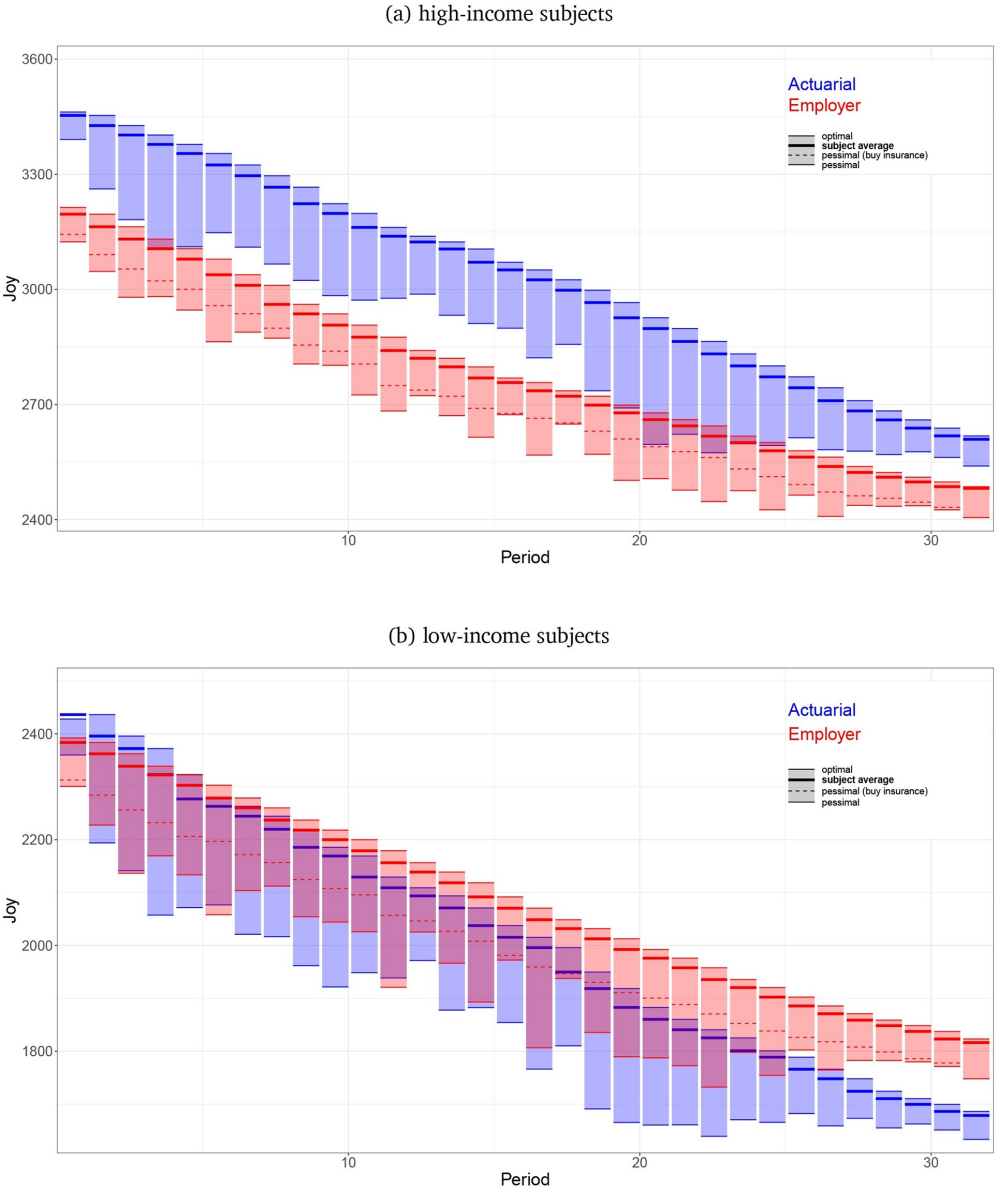
Joy earned by subjects compared to optimal. Includes panels: (a) High-income subjects, (b) Low-income subjects.

Fig 5 show the implications of subjects’ lack of investment in their future, plotting Joy lost relative to optimal, and how those losses accrue across life. Results for high- and low-income subjects are presented in Panels a and b, respectively. Within each panel, the blue bars plot actuarial values and the red bars plot employer values. For each subject in each period of all experiments, we used the DP to calculate the best and worst decisions that the subject could make in her effort to maximize joy over the remainder of her life, taking into account her current health and savings. Including the total joy actually earned in all previous periods, the top of each period-bar (‘optimal play’) shows the mean total lifetime joy that subjects could obtain by making the best possible decision in that period and then playing optimally for the remainder of life. The bottom of each period-bar (‘pessimal play’) shows the mean total lifetime joy that subjects could obtain by making the worst possible decision in that period but then playing optimally for the remainder of life. The bold line within each period-bar corresponds to the mean observed actual play. The total height of the period-bar shows the loss in lifetime joy between making the best and worst decisions that period. Finally, the dotted lines in the red employer bands show the higher mean expected aggregate joy with a pessimal decision, conditional on subjects always buying subsidized insurance.

Subjects’ decision efficiency –the bold line position within the bar– is relatively constant and much closer to optimal than pessimal, but by the end of life a significant proportion of potential lifetime joy has been lost to compounding small errors. (Aggregate attainable lost joy is ∼ 25% for high income, actuarial subjects, ∼ 22% for high income, employer subjects, ∼ 32% for low income actuarial subjects, and ∼ 23% for low income employer subjects.) As life progresses potential aggregate joy in the actuarial treatment decreases more than in the employer treatment. This impact is much larger for low-income subjects for whom the actuarial bars start above the employer bars but quickly fall and stay below, revealing the greater cost of non-optimal decisions. The height of the vertical period-bars is greater for the actuarial treatment than for the employer treatment for both income levels, indicating a greater potential for costly errors in decision making, and this comparative difference becomes even more exaggerated if the bottoms of the employer period-bars are limited by the dashed (buy employer insurance) decision.

The under-investment in the future and over-investment in current consumption had greater impacts on total joy obtained in the actuarial than in the employer treatment. Investments in savings and resilience are more critical to obtaining joy in the actuarial treatment: savings, because insurance costs increase in the second half of life, when the shock probability becomes higher; and resilience because insurance premiums vary negatively with investments in resilience. The flat lifetime rate for insurance in the employer treatment both obviates the need for significant savings and provides little incentive for investments in resilience. The optimal strategy in the employer treatment varied relatively little across life, with two exceptions. The first eluded most subjects, and was their most costly deviation; not spending more to increase health in the first few periods. Once the higher health was achieved, spending could be reduced to maintain that level. The other was to shift some health spending to joy at the end of life, which the subjects did to some extent. In contrast, decision in the actuarial environment were more complicated; optimal contributions change dynamically across life and necessitated weighing inter-temporal trade-offs across multiple interdependent investments. Optimal savings would require calculating and comparing the expected marginal value of spending in each period. To recognize the value of resilience, subjects needed to aggregate its multiple returns. Higher resilience meant higher health in the event of shock. That had an immediate return in more joy as well as less costly recovery and more income. The latter two benefits also had value that compounded over all future periods. This environment combined with subjects’ present bias in decision making had deleterious welfare.

The greater deviation between the predicted and observed behavior in the actuarial treatment suggests that its increased complexity, reduces subjects’ abilities to optimize their choices. Our findings are also consistent with research finding poor public comprehension of health insurance a strong preference for simpler plans [28]. Of particular significance, is that the individual responsibility required by the actuarial plan was particularly detrimental to the lower income subjects. Our results are consistent with a related literature, originating with Iyengar and Lepper [29] and summarized by Chernev et al [30], showing the number of choices negatively impacts on quality of choice. It is also consistent with the literature on laboratory experiments in which individuals must make dynamic decisions in multi-period lives [31–34]. Given that less than 3% of Americans follow four major guidelines for healthy behavior [35], our general findings are consistent with empirical patterns observed in the real world.

We note that our subjects in the low-income actuarial treatment purchased more insurance than those with higher income, as predicted by the DP optimizing agents. This apparently reflects a greater gain from buffering risk as income and health decrease with age in our environment. This experimental result seems somewhat contrary to observational field data findings in which low-income individuals are less likely to be insured. [36]. However, Finkelstein et al. [37] argue that in addition to the cost of providing coverage to purchaser insurance prices include subsidies for the care of uninsured individuals, and that this drives a wedge between price charged and the value to low-income consumers. The result is relatively low insurance take-up. Finkelstein et al. describe the evidence as “suggestive but indirect”(p. 275) and identify it as an important area for additional research. We also note they do not directly address why this impacts low-income consumers and not others. They offer two other possible reasons insurance might not be purchased, liquidity constraints and behavioral biases. The parameters in this environment did not have the large mark-up to create subsidies, nor did it have incomes low-enough to present liquidity constraints. Our environment reduced behavioral biases subject had a salient reminder (the shock wheel) of an unambiguous risk of shock of a known size. The environment and parameters could be altered to specifically test each possible reason.

Though low-income subjects insured at higher rates, they maintained lower resilience relative to high-income subjects, so they were correctly allocating given their variation in marginal benefits. Impediments to the purchase of health insurance, such as strong financial constraints, income interruption, under-forecast expenditures, and difficulties interacting with governmental agencies, may explain SES effects on health insurance coverage than insensitivity to risk. Such effects may force lower income people to insure through informal mechanisms, even at considerable expense, to prevent the extremely harsh consequences that a negative health shock would cause them [38].

Our finding of small differences in resilience investments across insurance type is consistent with much of the observational and quasi-experimental research which generally has found that the little to no *ex ante* moral hazard. However unlike observational studies, most of which depend on self-report to measure behaviors, our resilience measure could not be distorted by subject reporting, so our results are a valuable buttress to previous evidence.

The limitations of our study should be considered in interpreting these results. First, virtual ‘health’ in an experimental environment may be experienced very differently than real-world health. Behaviors, such as diet, exercise, and tobacco, alcohol and drug use, may be experienced and motivated differently than moving levers to pay for improved health in an economics laboratory. What is very encouraging, however, is that our subjects’ behavior matched most of the qualitative predictions of the DP optimizing agents, and deviations from optimal behavior, such as under-investment in health and resilience throughout life, are commonly observed in the real world.

Another limitation is that actuarial subjects in our experiment were not precisely informed about how insurance price was calculated: they were simply told they would have the option buy insurance and their current price would be shown on their screen. However, from period to period subjects could easily see that insurance price increased/decreased with the rise and fall of their investment in resilience.

All subjects were endowed an initial health stock of 70/100 in their first period of life. We knew that in all cases this was below the level optimizing agents would hone in to. Many subjects tended to maintain this initial stock with small deviations (±5) from period to period until the endgame in which they allowed it to deteriorate. It is unclear whether they would have maintained a higher, ‘near optimal’ level of 75-80 if that were their initial allocation, or whether and how quickly they would have allowed their health to deteriorate if their initial stock was higher than optimal. However, the experiment had three training lives and chat groups with complete public information on like-subjects’ behavioral choices. This allowed the subjects to jointly explore their strategy space, prior to the 32 period lifetimes that generated our data. These factors suggest that the behavioral proclivities we observed are not due to an anchoring effect.

Assessments of the experimental environment’s validity with respect to actual health behavior will be necessary for evaluating the productivity of this approach. Two future experiments we plan to implement to test validity are: 1) perform multi-day experiments that substitute real-effort, accelerometer-measured exercise, for investments in resilience while maintaining other features of the current experimental protocol; and 2) conducting the experiments with other ethnic and SES groups as opposed to university students to evaluate the generality of our findings.

## Conclusion

Our results have theoretical, policy and methodological implications. Even though under optimal play the actuarial plan had higher potential life satisfaction, we find that it did not outperform the employer plan in our experiments. The gains in efficiency due to individual responsibility under the actuarial plan were very modest for high-income subjects and non-existent for low-income subjects. All actuarial subjects under-invested in savings and resilience to shocks, compared to optimizing agents. Low investment in resilience (fitness) also observed in the actuarial treatment suggests that concerns about moral hazard are overemphasized in policy debates. On the other hand, the employer plan resulted in a simpler decision space and significantly reduced observed disparities due to income level and phase of life. Thus, in this environment, the advantages of risk-sharing and income transfers appear to outweigh the gains from incentives for individual responsibility. Might a hybrid system such as: an employer-based system with appropriate premium incentives for healthy behavior outperform the two systems we investigated here?

The findings from this experiment illustrate the benefits of the experimental laboratory, as a complement to existing research methods. This method allows us to simulate a complex, multi-variate dynamic health decision environment and assess the lifetime population effects that might be imposed by individual health behaviors. At the same time, it permits controlled experimental manipulations of policy alternatives where evaluation is not impeded by selection biases. A major challenge that confronts this experimental approach is whether it can be applied with validity to real-world health behavior. Validity assessment is a clear next step, but the results obtained so far are encouraging as they qualitatively match observed real behavior.

There is ample scope for mutual translation from such experiments to observational research and real policy manipulations. Hypotheses generated from population-based studies could be tested with controlled laboratory experiments, and findings from laboratory studies can motivate new analyses of population data. Such experiments can be used to explore whether policies under consideration will result in expected effects or have untoward and unexpected consequences. While Miller et al. [16], and Borgschulte and Vogler [17] convincingly show that the ACA Medicaid expansion saved lives, lack of conclusive benefits from prior expansions contributed in part to some states opting out of ACA Medicaid expansion. Lab experiments can be useful tool to develop evidence and speed adoption of policies that save lives. Similarly, the results of existing policy experiments, such as Romneycare and the ACA, can inform new experimental treatments to isolate causality and to assess modified policy alternatives.

More broadly, the experimental environment can be adapted to address other issues in which observational studies were unable to reach conclusive findings due to insufficient data or co-linearity of important variables. There is the potential to disentangle, for example, the relationship between education and health, as well as conduct experiments addressing chronic disease and how social norms and learning impact health decisions. These factors potentially interact with insurance policy. We advocate that experimental test environments like ours are a low-cost alternative to understanding these nuances of health behaviors before proceeding to much more costly field studies and wholesale policy changes. Furthermore these environment provide results quickly.

## Supporting information

S1 File (PDF)
