# Supplementary material for: An investigation of health insurance policy and behavior in a virtual environment
Source: PLoS One. 2021 Apr 6;16(4):e0248784. doi: 10.1371/journal.pone.0248784 (PMC8023465; doi:10.1371/journal.pone.0248784)
Supplement: S1 File — (PDF) [file pone.0248784.s001.pdf]

Summary statistics

S1 Table presents the descriptive statistics regarding subject decisions and outcomes for the four experimental treatments in the first and second halves of life.

S1 **Table.** Summary statistics

	Low income				High income			
	Actuarial		Employer		Actuarial		Employer	
	Young	Old	Young	Old	Young	Old	Young	Old
Insurance purchase	0.61	0.60[a]	0.94	0.98[a]	0.40	0.51[a]	0.88	0.94[a]
Resilience	0.78	1.48	0.07	0.15	0.86	1.48	0.15	0.33
Health	63.91	56.16	64.42	57.61	65.36	60.05	63.79	60.13
Savings	15.92	12.46	9.91	6.11	21.04	16.29	5.51	5.00
Joy	79.75	82.16	79.75	82.16	79.75	82.16	79.75	82.16
Income	102.31	94.79	83.33	78.09	129.52	124.64	103.33	100.63
Average lifetime	29.4		31.9		31.4		31.1	
Premature deaths	26		5		9		7	

[a] Excluding last period.

Transfers

S2 Table provides calculations of the transfers in the employer based insurance. Profit (π) per period for each group of subjects is the Insurer's "Payout" less Withheld (from subjects' income) and Premium paid. Sum-zero subtract one quarter of \$6.43 of insurer profit from each group. This column sums to zero and is the inter-group transfers. Mean transfers from young to old are \$7.65, and \$3.60 from high- to low-income.

S2 **Table.** Transfer calculations

Group	Payout	Withheld	Premium	$\pi/period$	Sum zero
Low inc. young	21.54	19.43	8.46	-6.34	-4.74
Low inc. old	36.51	17.73	8.46	10.32	11.93
High inc. young	19.43	23.7	7.9	-12.17	-10.56
High inc. old	32.26	22.45	8.05	1.76	3.37
Total	109.75	83.31	32.87	-6.43	0

Supplemental regressions

S3 Table report coefficient estimates and level cut points from ordered probit regressing Resilience on insurance type, as robustness check to the regression on resilience as continuous variable, reported in the main text. Estimates are consistent with the continuous version. In all cases employer insurance reduces resilience.

S3 Table. Estimates from ordered probit regressing resilience on insurance type

	(1)<br>Young<br>low income	(2)<br>Young<br>high income	(3)<br>Old<br>low income	(4)<br>Old<br>high income
Employer	-1.217***	-1.283***	-1.090***	-1.154***
	(0.0971)	(0.100)	(0.110)	(0.0861)
/cut1	0.408***	0.0527	0.181*	-0.216***
	(0.0579)	(0.0770)	(0.0940)	(0.0799)
/cut2	0.708***	0.259***	0.638***	0.0874
	(0.0617)	(0.0764)	(0.0856)	(0.0579)
/cut3	1.232***	0.662***	1.148***	0.606***
	(0.0853)	(0.0518)	(0.0905)	(0.0786)
/cut4	1.647***	1.094***	1.850***	1.286***
	(0.0954)	(0.0575)	(0.136)	(0.110)
Observations	4,224	4,224	4,096	4,096

Robust standard errors in parentheses

*** p<0.01, ** p<0.05, * p<0.1

Results summary

All treatment effects, for both optimally computed (DP maximizers) and observed (human subjects), are summarized in the following tables: In both tables, sign denotes direction of effect, number of signs denotes size of mean effect relative to its range and variation. Blank cells indicate no meaningful effect. Survival is calculated only for the old. S4 Table reports difference between DP joy maximizers and the human subjects.

S4 Table. Effect size, (DP maximizers - human subject)

Income type	Ins. type age	Insurance purchase	Resilience investment	Health investment	Survival to period 32	Total savings	Total jjoy consumed
Low	Act/Yng	+	-	+		+++	+++
	Act/Old	- -	++	++	+++	+++	+++
	Emp/Yng			+		+	++
	Emp/Old			++	+	+	++
High	Act/Yng		++	+		+++	++
	Act/Old	- -	+++	++	+	+++	++
	Emp/Yng	-				++	++
	Emp/Old				+	++	++

Legend: sign notes direction. -/+ small but meaningful,- -/ ++ moderate, - - -/+++ large difference

S5 Table has three sections. In each section, there is a column for the within DP joy maximizer comparison and a column for the within human subject (HS) comparison. The top section reports the differences between the employer and actuarial treatments.

The middle section reports the differences between old and young. The bottom section reports the difference between high- and low-income.

S5 **Table. Effect size for all treatment comparisons**

		Insurance purchase		Resilence		Health		Survival		Savings		Joy	
Compare	Subgroup	DP	HS	DP	HS	DP	HS	DP	HS	DP	HS	DP	HS
Emp-Act	Yng low	++	++	-	-		+			- -	-		+
	Old low	+++	++	- -	-		++		+++	- -	-	-	-
	Yng high	+++	++	- -	-		-			- -	-	-	-
	Old high	+++	++	- -	-				+	- -	-	- -	- -
Old-Yng	Act low	- - -		++	+	-	- -	[redacted]		-		++	++
	Act high	- -	+	++	+	-	- -					+	+
	Emp low			++			-					+	+
	Emp high			++	+		-						+
High-Low	Act yng	- -	- -	+++	+	+	+			+	+	+++	+++
	Act old		-	++	+	+	++		+++			+++	+++
	Emp yng		-		+	+	+			+	-	+++	+++
	Emp old		-	+	+	+	+		-	+	+	+++	+++

Legend: sign notes direction. -/+ small but meaningful, - -/ ++ moderate, - - -/+++ large difference

Decomposition

Moral hazard and joy transfers – Subjects under the Employer treatment (especially Low-income) were able to perform relatively better, compared to maximizers, than their actuarial counterparts. This indicates that the subsidies and implicit transfers provided under the employer plan, from high to low-Income and from young to old subjects, are having a marked effect even though all subjects tend to underinvest in resilience. Because actuarial insurance bases its premiums on individual subject resilience and health, there is by definition no moral hazard and no welfare transfers between categories of actuarial subjects. But considering the degree to which the actuarial subjects fall 'behaviorally' short of optimal resilience investment, we can assume that that employer subjects underinvesting to any greater extent are falling subject to moral hazard.

The mean percentage by which actuarial subjects underinvest in resilience was 54%. Premiums in the employer insurance treatment were pre-calibrated to be profitable despite anticipated underinvestment in resilience. To measure subject-induced moral hazard under the employer treatment, we simply calculated the insurer's expected profit and the subjects' expected joy for every level of health and resilience the subject could have had. We then calculated how much the insurance company could have transferred to the maximizer/subject conditional on her investing in each resilience level in order for the insurer to have the same expected profit as it does when subjects have a fitness of 0. Finally, we calculated how much more expected joy the subject could have earned with the conditional transfer. We defined potential moral hazard costs as the gain in expected joy between a subject's optimal resilience level and observed (mean) levels in actuarial subjects. We then calculated how high expected joy level would have been if there were transfers and the subjects maintained the fitness level that actuarial subjects maintained, which was 46% of the potential moral hazard cost. The balance, 54% of the transfer benefits, can be attributed to the behavioral proclivity to underinvest in resilience, even when there are incentives to invest, as in the actuarial treatment.

S6 **Table. Employer subjects' decomposition of joy obtained**

	Low income		High income	
	DP	Subjects	DP	Subjects
**Actuarial joy**	**2423**	**1679**	**3462**	**2609**
Selection bias	0	0	0	0
Transfer young → old	-31	4	-25	1
Transfer high → low	99	66	-75	-112
Admin fees	-27	-21	-37	-14
Moral hazard	-80	-57	-96	-63
Health modified joy	0	27	0	23
Death prevention joy	0	173	0	11
Total adjustments	-39	193	-233	-154
(% Actuarial joy obtained)	(-1.6%)	(+11.5%)	(-6.7%)	(-5.9%)
Residual joy	-21	-56	-16	27
(% Actuarial joy obtained)	(0.3%)	(-8.2%)	(-0.5%)	(+4.9%)
**Employer joy**	**2391**	**1816**	**3213**	**2482**

Using this information and mean expenditures on the various possible investments, S6 Table below decomposes the mean components of joy obtained under the employer treatment, relative to the actuarial treatment, by considering the insurance subsidies provided and received.

The first line of S6 Table shows the mean joy amounts at the end of life for the each income group in the actuarial Treatment. The last line shows the corresponding means in the employer treatment. Each intermediary line is the estimated impact of one difference between treatments. We saw no selection bias in the employer treatment, neither in the DP nor human subjects, meaning young and old purchase insurance at near identical rates. The third line reports estimates for the benefit of consumption smoothing from the transfers from young to old netted across life. The human subjects benefit mildly from these. The DP is actually hurt by the transfers. The DP has the foresight to save to smooth consumption, so the imposed transfers constrain its ability to adjust savings according to the stochastic shock pattern experienced. The fourth line reports the estimated impact of the transfers from rich to poor: the effects are strong and all in the correct direction. The administration fee line is the estimation of the cost in joy to the employer subjects of the profit the insurer extracts. Moral hazard is the estimated impact on joy of maintaining resilience at a lower level, which may be individually optimal but socially costly. The Health Modified Joy line is the estimate of how much higher health in the Employer versus actuarial treatment benefits subjects by increasing their joy: DP maximizers do not display this effect. The Death Prevention Joy line is the estimate of how much extended life in the employer versus actuarial treatment benefits subjects by increasing their joy: again, DP maximizers do not display this effect. The Residual Joy is the unexplained difference between observed joy in the actuarial and employer treatments after accounting for the Total Adjustments.

Subjects - The young to old transfer was estimated by averaging the consumption across the young and old periods, and taking the difference between the smoothed and unsmoothed consumption. Admittedly, this is only part of the benefit of the transfer—the amount applied to consumption. The other part is the benefit gained by applying some of the transfer to increasing health. This is the health modify joy line, which is the difference in the amount of joy actuarial consumption would have resulted

in between employer treatment levels of health and the level in the actuarial treatment. The rich to poor transfer was calculated by taking the difference between consumption if was increased by (transfer + mean income)/mean income. Admin was found by the same method as the previous transfer but with the amount of the increase in profit in the employer treatment relative to the actuarial. Prevent death is the difference in expected mean joy at the end of life including or not including subjects who died early.

DP - We calculated for the employer subjects net payments to the insurer, summing withholdings and premium paid less health regenerated from insurance, for each age and income combination. The administration cost is the adjustment required for the employer plan to have the same profit as the actuarial plan, \$-0.53 per subject per period. For the administration line we reran the DP adjusting the income each period by this amount. The young to old and low to high transfer lines follow the same logic. After adjusting for the difference in administration costs, we found the mean for age or income and used that amount to adjust the DP's income. We report the difference in Joy earned with adjusted and unadjusted income. The Joy Modified income line is not calculated for the DP, because using method above the DP increases it income so this is already included. The prevent death line is not calculated because there were no lives under either treatment in which the DP died prematurely.
